# First person – Luiza Coelho

**DOI:** 10.1242/dmm.053198

**Published:** 2026-09-30

## Abstract

First Person is a series of interviews with the first authors of a selection of papers published in Disease Models & Mechanisms, helping researchers promote themselves alongside their papers. Luiza Coelho is first author on ‘ Preserved satellite cell pool in a laminin-α2-deficient model of congenital muscular dystrophy’, published in DMM. Luiza conducted the research described in this article while an undergraduate student in Mariz Vainzof's lab at University of São Paulo, São Paulo, Brazil. She is now a Master's student in the same lab, investigating muscle regeneration in health and disease and the factors that modulate this process.

**Figure:**
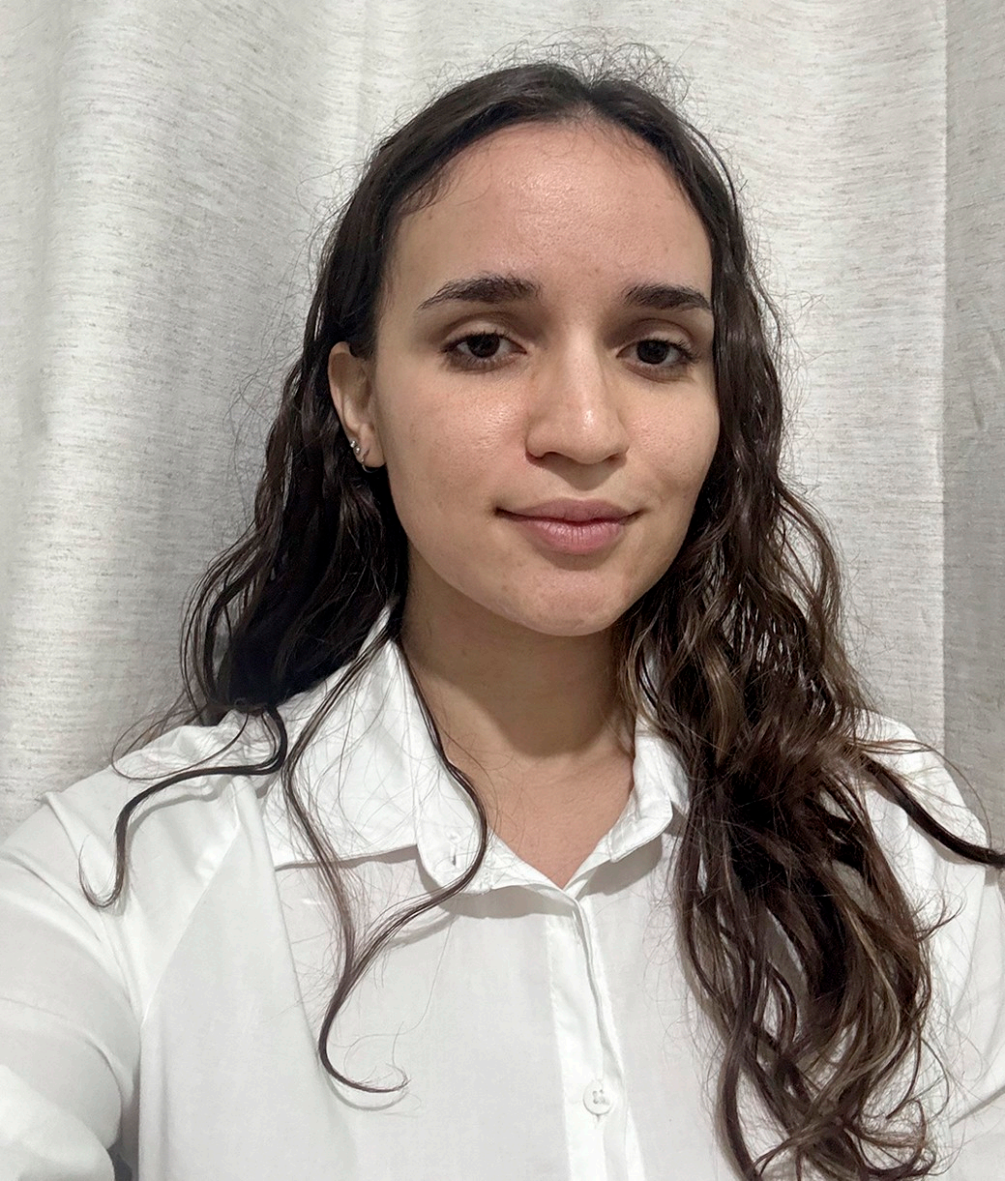
Luiza Coelho

**Who or what inspired you to become a scientist?**

Understanding the nature of science is what inspired me to become a scientist. I realize that even though science is not completely unbiased, since it is a human endeavour, it is highly reliable due to its transparency. This transparency allows it to be questioned, criticized and reproduced by other scientists. Therefore, the participation of people from diverse backgrounds is crucial, as different perspectives strengthen science and drive its advancement.

**What is the main question or challenge in disease biology you are addressing in this paper? How did you go about investigating your question or challenge?**

The main question we address in this paper is whether the failure of muscle regeneration during disease progression in the dy^2J^ mouse, a model for congenital LAMA2-related muscular dystrophy, is due to satellite cell (SC) depletion. To investigate this, we analysed the presence, activation and differentiation of SCs in the gastrocnemius muscle across different ages in the dy^2J^ model using immunofluorescence and real-time PCR techniques.

**How would you explain the main findings of your paper to non-scientific family and friends?**

Muscles have a great regenerative capacity, which is driven by muscle stem cells. However, in patients and mice with muscular dystrophy (a group of muscle diseases), this regeneration is not enough to compensate for the muscle damage caused by the illness. In our study, we investigated whether muscle stem cells are still present and active throughout disease progression in the dy^2J^ mouse. We found that these stem cells are present at all stages of the disease, but they seem to be unable to properly proliferate and regenerate muscle tissue. This inability leads to progressive muscle loss as the disease advances.

**What are the potential implications of these results for disease biology and the possible impact on patients?**

Our results show that the progressive failure in muscle regeneration is not due to SC depletion, as previously hypothesized. Furthermore, our findings corroborate evidence that laminin-α2 is crucial for SC proliferation. Collectively, this knowledge is valuable for developing future treatment strategies, suggesting that therapeutic efforts could focus on using substances to induce endogenous SC proliferation rather than relying on stem cell transplantation in patients.

Our results show that the progressive failure in muscle regeneration is not due to [satellite cell] depletion, as previously hypothesized

**Figure:**
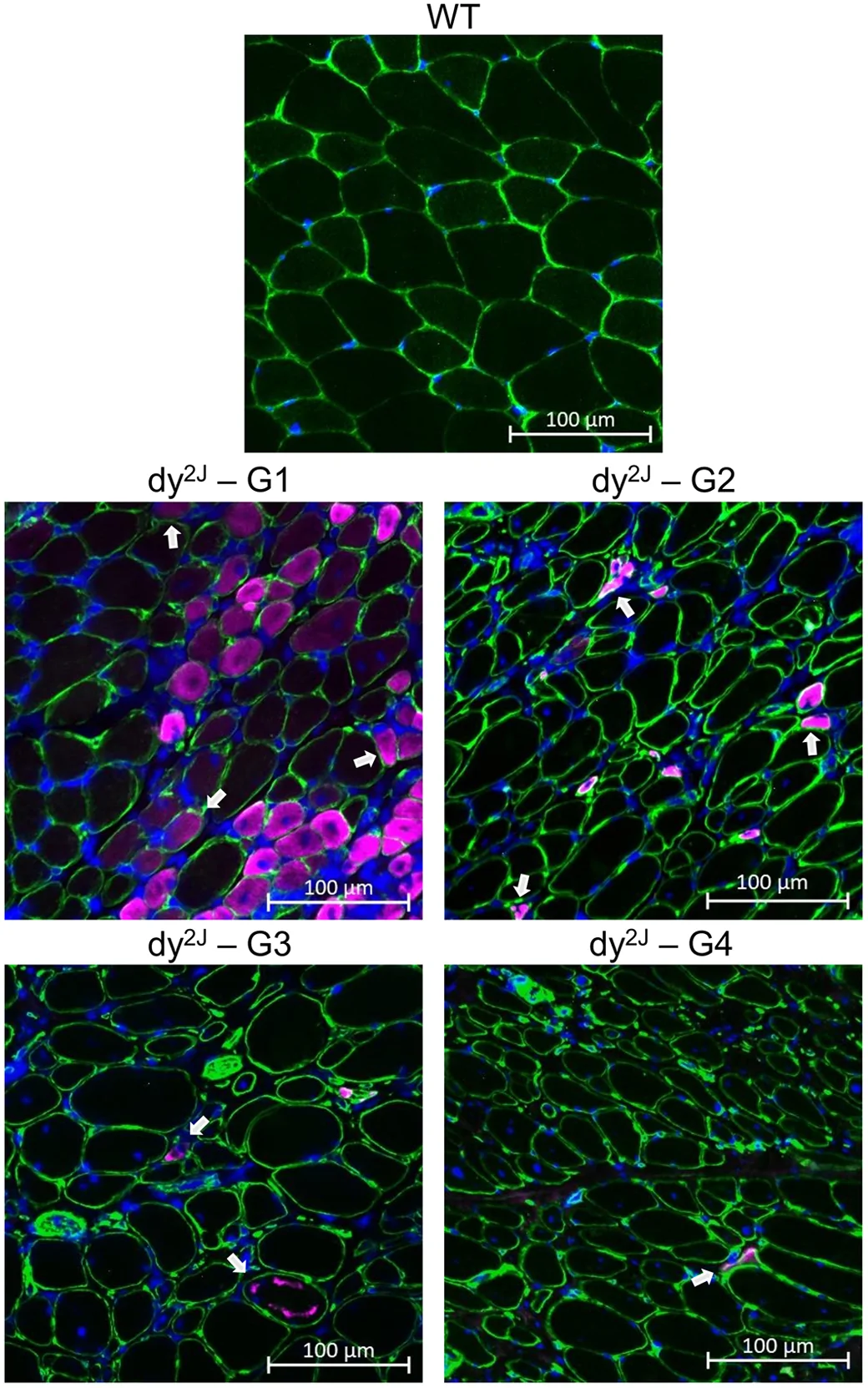
Immunofluorescence showing the progressive decline of newly regenerated muscle fibres (in magenta) across different disease stages in the dy^2J^ mouse model of muscular dystrophy.

**Why did you choose DMM for your paper?**

I chose DMM because it is a great reference for anyone working with disease models. Since my research focuses on understanding muscular dystrophy using the dy^2J^ mouse, DMM felt like the most natural fit to share our findings with the right community. Besides that, the journal's open-access policy and the fact that it is published by The Company of Biologists, a not-for-profit organization, were essential to our decision, as I strongly believe that making scientific knowledge accessible is key to the advancement of science.

**Given your current role, what challenges do you face and what changes could improve the professional lives of other scientists in this role?**

My biggest challenge is securing adequate funding for scientific research. Purchasing reagents and equipment can often be difficult due to limited financial resources. Furthermore, researchers are frequently underpaid. As a result, even though Brazil produces highly qualified scientists in its universities, they often leave the country in search of better career opportunities, representing a significant loss for our nation. Therefore, increased funding for science, not only to better compensate researchers but also to acquire essential materials, is crucial to improve the professional lives of scientists.

**What's next for you?**

I am currently pursuing a Master's degree in Biological Sciences, investigating muscle regeneration in dy^2J^ mice. Using primary myoblast cultures, I aim to identify the cellular and molecular mechanisms altered in this model.

**Tell us something interesting about yourself that wouldn't be on your CV**

I enjoy creating closed plant terrariums from recycled glass and plastic, often giving discarded laboratory containers a new life.
